# Red Blood Cell Distribution Width as a Predictive Marker for Coronary Artery Lesions in Patients with Kawasaki Disease

**DOI:** 10.1007/s00246-021-02633-x

**Published:** 2021-05-25

**Authors:** Li Ming, Hui-ling Cao, Qiushu Li, Gengsheng Yu

**Affiliations:** 1 Chongqing Key Laboratory of Pediatrics, China International Science and Technology Cooperation Base of Child Development and Critical Disorders, Department of Heart Centre, National Clinical Research Center for Child Health and Disorders, Ministry of Education Key Laboratory of Child Development and Disorders, Children’s Hospital of Chongqing Medical University, Chongqing, 400014 China; 2 Chongqing Key Laboratory of Pediatrics, China International Science and Technology Cooperation Base of Child Development and Critical Disorders, Department of Neonatology, National Clinical Research Center for Child Health and Disorders, Ministry of Education Key Laboratory of Child Development and Disorders, Children’s Hospital of Chongqing Medical University, Chongqing, China

**Keywords:** Kawasaki disease, Coronary artery lesion, Red blood cell distribution

## Abstract

This study aimed to investigate the association between red blood cell distribution width (RDW) and the risk of coronary artery lesions (CALs) in patients with Kawasaki disease (KD). A total of 1355 patients who met the diagnostic criteria for KD were reviewed between January 2018 and December 2019, including 636 patients with CALs and 719 patients without CALs. Blood samples for RDW were obtained at admission (before intravenous immunoglobulin treatment). A logistic regression analysis was performed, and a receiver operating characteristic curve was constructed to determine the prognostic value of RDW standard deviation (RDW-SD) and RDW coefficient of variation (RDW-CV). The study was registered at www.chictr.org.cn, No.: ChiCTR 2000040980. The results showed that RDW-SD increased in patients with complete KD and CALs compared with patients with complete KD without CALs (39 fL vs. 38 fL, respectively; *p* = 0.000). RDW-CV in patients with complete KD and CALs was significantly higher compared with patients with completed KD without CALs (*p* = 0.000). Further multivariate logistic regression analysis revealed that RDW-SD was an independent marker of CALs in patients with complete KD (*p* = 0.001), but no association was found between RDW-CV and CALs. The area under the curve of RDW-SD for predicting CALs in patients with complete KD was 0.606 (95% confidence interval 0.572–0.640; *p* = 0.000) with a sensitivity and specificity of 61% and 55%, respectively, when the optimal cut-off value of RDW-SD was 38.5 fL. RDW-CV increased in patients with incomplete KD and CALs compared with patients without CALs (13.55% vs 13.3%, respectively; *p* = 0.004), and multivariate logistic regression analysis revealed that RDW-CV was an independent marker of CALs in patients with incomplete KD (*p* = 0.021). The area under the curve of RDW-CV for predicting CALs in patients with incomplete KD was 0.597 (95% confidence interval 0.532–0.661; *p* = 0.004) with a sensitivity and specificity of 40% and 77%, respectively, when the optimal cut-off value of RDW-SD was 13.85%. Conclusion: RDW can be used as an independent predictive marker of CALs in patients with KD, but the type of KD should be considered. RDW-SD was an independent marker of CALs in patients with complete KD, while RDW-CV was a predictor of incomplete KD.

## Introduction

Kawasaki disease (KD), which is an acute systemic vasculitis of unknown cause, usually occurs in children < 5 years of age [1]. The most serious complications of KD are coronary artery lesions (CALs), such as dilatation and aneurysm. The prevalence of CALs can be minimized if treatment with intravenous immunoglobulin (IVIG) is administered before the 10th day of disease [2]. However, approximately 10–15% of patients with KD are IVIG resistant and are thus at an increased risk of developing CALs [3, 4]. CALs may persist and progress to obstruction or stenosis, leading to ischemia and even sudden death. Therefore, KD is the leading cause of acquired heart disease in developed countries [5, 6]. The prognosis, follow up, and long-term management of patients with KD depend mainly on the severity of CALs. Clinicians tend to treat patients with KD who are at a high risk of CALs more aggressively to block the inflammatory response and minimize progression to CALs. Although risk factors for CALs have been studied extensively, no specific biological markers for predicting CALs in clinical practice have been established thus far.

Red cell distribution width (RDW), which is a marker of variation in the size of circulating erythrocytes (anisocytosis), is routinely measured in clinical practice as part of the complete blood count (CBC). Traditionally, RDW is used in the differential diagnosis of anemia [7]. In 2007, Felker et al. [8] first discovered that an increase in RDW is an independent predictor of morbidity and mortality in patients with chronic heart failure. Growing interest has been expressed in the clinical value of RDW. An increasing number of studies have demonstrated that a higher RDW is a prognostic factor for various cardiovascular system diseases, such as coronary artery disease [9], hypertension [10], heart failure [7, 8, 11], peripheral artery disease [12], coronary artery ectasia [13], and congenital heart disease [14]. However, studies on the relationship between RDW and CALs in patients with KD are limited, and these studies mainly focus on the RDW coefficient of variation (RDW-CV). RDW-CV is calculated by dividing the standard deviation (SD) of red blood cell (RBC) volume by mean erythrocyte volume and multiplying by 100% to express the result as a percentage [15]. Because information might be lost by expressing RDW relative to MCV, Hoffmann et al. [16] proposed that future studies on the prognostic value of RDW should focus on RDW-SD. Tonelli et al. [17] suggested that RDW-SD may be more useful as a prognostic marker than RDW-CV in the general population. Therefore, in the present study, we aimed to investigate the relationship between RDW, including RDW-CV and RDW-SD, and CALs in patients with KD.

## Materials and Methods

### Patient Selection

Between January 2018 and December 2019, this retrospective study was performed on 1355 patients with KD admitted to the Children’s Hospital of Chongqing Medical University. The inclusion criteria were as follows: (1) ≤ 18 years of age; (2) acute disease stage; (3) diagnosis of KD according to the criteria established by the American Heart Association. The exclusion criteria were (1) incomplete clinical data; (2) IVIG treatment at other medical institutions before admission/no IVIG treatment during hospitalization; (3) congenital heart disease, cardiomyopathy, myocarditis, valvular heart disease, severe arrhythmia, heart failure, or other heart disease; (4) moderate-to-severe anemia, leukemia, multiple myeloma, myelodysplastic syndrome, or other blood system disease.

The present study protocol was reviewed and approved by the Ethics Committee of the Children’s Hospital Affiliated to Chongqing Medical University, and with the its approval, this study required no conformed consent. All methods were performed in accordance with Declaration of Helsinki and the relevant guidelines.

### Data Collection

Demographic characteristics, laboratory data, and echocardiographic data were collected. Laboratory tests, including white blood cell counts, platelet counts, hemoglobin, mean erythrocyte volume, RDW-CV, RDW-SD, neutrophil percentage, lymphocyte percentage, eosinophil percentage, erythrocyte sedimentation rate, serum C-reactive protein, procalcitonin, glutamyltranspeptidase, alanine transaminase, aspartate aminotransferase, total bile acid, total bilirubin, albumin, uric acid, creatinine, serum sodium, fibrinogen, and activated partial thromboplastin time were measured in all subjects before IVIG treatment. RDW-CV and RDW-SD were determined using the XS-800i (Sysmex, Kobe, Japan) automated hematology analyzer. The normal range of RDW-CV (%) in our laboratory was less than 15.5%, and the normal range of RDW-SD was between 40 and 80 fL.

Echocardiography was used to detect CALs during hospitalization. Due to lack of height data, no mean body surface area (BSA)-adjusted Z-score was available. CALs were defined according to an internal lumen diameter of > 2.5 mm in patients < 3 years of age, > 3 mm in patients aged 3–9 years, and > 3.5 mm in patients aged 9–14 years; an internal segment diameter ≥ 1.5 times that of an adjacent segment; and an irregular lumen [18].

### Statistical Analysis

Continuous variables are expressed as mean ± standard deviation or median (25th–75th percentile) if non-normally distributed and were compared using independent-samples *t* tests or Mann–Whitney *U* tests between study groups. The normality of distribution of variables was checked using the Kolmogorov–Smirnov test. Categorical variables are expressed as percentages and were analyzed with the Chi-squared test. To evaluate the independent effects of RDW on CALs in patients with KD, a multivariate logistic regression analysis was performed. This analysis included age, sex and variables with a *p* value of < 0.05. The area under the receiver operating characteristic (ROC) curve (AUC) was analyzed to assess the predictive accuracy of RDW for CALs and to identify the optimal cut-off point. SPSS 25.0 (IBM Corporation, Armonk, New York, USA) was used for statistical analysis. A *p* value < 0.05 was considered statistically significant.

## Results

A total of 1355 patients (856 males and 499 females) were enrolled in this study. The ages ranged from 2 to 184 months (median age: 28 months). Two hundred and ninety-five of those (21.8%) had incomplete KD and six hundred and thirty-six developed CALs despite receiving IVIG.

### Characteristics of Patients with Complete KD and Incomplete KD

We performed a subgroup analysis of the types of KD. In the subgroup of patients with complete KD, compared with the group without CALs, RDW-CV, RDW-SD, glutamyltranspeptidase, procalcitonin, male sex and the frequency of IVIG resistant were significantly higher in patients with CALs (Table 1). On the contrary, hemoglobin, AST/ALT, albumin, and serum sodium were significantly lower in patients with CALs (Table 1). In the incomplete Kawasaki disease subgroup, male sex and RDW-CV levels in the CALs group were significantly higher than those in the without CALs (Table 2). While mean erythrocyte volume and total bilirubin were significantly lower in patients with CALs (Table 2).

**Table 1:** Demographic, laboratory characteristics of patients with complete Kawasaki disease

Variables	All (*n* = 1060)	CAL + (*n* = 482)	CAL − (*n* = 578)	*p* value
Age (month)	29 (17–46)	27 (17–42.25)	32.00 (17.00–48.00)	0.103
Male sex (%)	669 (63.1%)	326 (67.6%)	343 (59.3%)	0.005
IVIG resistance (%)	41 (3.9%)	26 (5.4%)	15 (2.6%)	0.019
White blood cell (109/L)	14.18 (10.88–17.78)	14.47 (11.1–18.29)	13.93 (10.82–17.50)	0.144
Platelet count (109/L)	385 (311–484.75)	382.5 (311.0–492.25)	387 (310.5–478)	0.879
Hemoglobin (g/l)	108 (101–114)	106 (99.0–113.0)	110 (104–115)	0.000
Mean erythrocyte volume (fL)	79.9 (77.7–82.2)	79.75 (77.6–81.9)	80.0 (77.9–82.4)	0.085
RDW-SD (fL)	39 (37–41)	39 (38–41)	38 (37–40)	0.000
RDW-CV (%)	13.3 (12.8–14)	13.5 (13.0–14.23)	13.2 (12.7–13.72)	0.000
Percentage of neutrophil	0.73 (0.62–0.82)	0.73 (0.62–0.82)	0.73 (0.63–0.82)	0.600
Percentage of lymphocyte	0.22 (0.14–0.31)	0.22 (0.14–0.32)	0.22 (0.14–0.31)	0.827
Lymphocyte/neutrophil	0.30 (0.17–0.50)	0.31 (0.17–0.51)	0.30 (0.17–0.49)	0.821
Percentage of eosinophils	0.02 (0.01–0.04)	0.02 (0.01–0.04)	0.02 (0.01–0.04)	0.203
Erythrocyte sedimentation rate (mm/L)	73.0 (54.0–90.0)	73.0 (54.0–90.0)	73.0 (55.0–89.0)	0.682
CRP ≥ 10 (%)	987 (93.1%)	451 (93.6%)	536 (92.7%)	0.628
Procalcitonin	0.547 (0.190–1.72)	0.72 (0.22–1.98)	0.46 (0.17–1.50)	0.003
Glutamyltranspeptidase (U/L)	33.0 (13.88–111.00)	43.0 (15.7–116.0)	27.0 (13.0–104.0)	0.005
Alanine transaminase (IU/L)	31.0 (16.9–77.9)	33.1 (17.0–74.2)	30.15 (16.83–84.68)	0.543
Aspartate aminotransferase (IU/L)	28.2 (22.3–41.1)	27.4 (22.0–40.0)	29.0 (22.63–44.50)	0.070
AST/ALT	1.00 (0.53–1.64)	0.92 (0.51–1.54)	1.07 (0.54–1.72)	0.043
Total bile acid (umol/L)	7.8 (4.5–16.5)	8.2 (4.7–17.2)	7.2 (4.2–14.9)	0.154
Total bilirubin (umol/L)	5.30 (3.30–8.70)	5.30 (3.30–8.40)	5.30 (3.40–8.80)	0.586
Albumin (g/L)	37.8 (34.5–40.7)	37.0 (33.5–39.8)	38.4 (35.60–41.20)	0.000
Uric acid (umol)	194 (154.18–244)	191.0 (150.0–241.75)	195.2 (155.6–247.0)	0.244
Creatinine (umol/L)	26 (22–31)	26.0 (22.0–32.0)	26.0 (22.0–30.0)	0.114
Serum sodium (mmol/L)	136.9 (135.1–138.8)	136.7 (134.9–138.4)	137.1 (135.2–139.0)	0.007
Fibrinogen (g/L)	6.25 (5.00–7.17)	6.24 (4.95–7.04)	6.27 (5.11–7.37)	0.122
Activated partial thromboplastin time (s)	30.1 (28.0–32.8)	30.3 (27.8–32.9)	29.95 (28.2–32.78)	0.624

*CALs* coronary artery lesions, *CI* confidence interval, *IVIG* intravenous immunoglobulin, *RDW-CV* Red blood cell distribution width variation coefficient, *RDW-SD* Red blood cell distribution width standard deviation, *CRP* C-reactive protein, *AST* Aspartate aminotransferase; *ALT* Alanine transaminase

**Table 2:** Demographic, laboratory characteristics of patients with incomplete Kawasaki disease

Variables	All (*n* = 295)	CAL + (*n* = 154)	CAL − (*n* = 141)	*p* value
Age (month)	28 (15–53)	26 (15.75–47.25)	34 (14.5–57.0)	0.253
Male sex (%)	187 (63.4%)	107 (69.5%)	80 (56.7%)	0.023
IVIG resistance (%)	4 (1.4%)	2 (1.4%)	2 (1.3%)	0.929
White blood cell (109/L)	12.29 (8.88–15.88)	12.2 (8.29–17.22)	12.35 (9.31–14.88)	0.651
Platelet count (109/L)	406 (309–540)	407 (285.25–568.75)	406 (324.5–513)	0.803
Hemoglobin (g/l)	108 (101–117)	108 (100.75–116.25)	110 (102–118)	0.273
Mean erythrocyte volume (fL)	79.9 (77.6–82.3)	79.5 (76.5–82.4)	80.5 (78.4–82.15)	0.022
RDW-SD (fL)	39 (37–41)	39 (37–41)	39 (37–41)	0.219
RDW-CV (%)	13.3 (12.8–14.2)	13.55 (12.9–14.3)	13.3 (12.7–13.8)	0.004
Percentage of neutrophil	0.63 (0.52–0.72)	0.61 (0.48–0.70)	0.65 (0.55–0.74)	0.150
Percentage of lymphocyte	0.30 (0.22–0.41)	0.32 (0.24–0.43)	0.29 (0.21–0.40)	0.069
Lymphocyte/neutrophil	0.47 (0.31–0.79)	0.52 (0.35–0.92)	0.44 (0.28–0.73)	0.112
Percentage of eosinophils	0.02 (0.01–0.04)	0.02 (0.01–0.04)	0.02 (0.01–0.04)	0.474
Erythrocyte sedimentation rate (mm/L)	66.5 (48.0–85.75)	65.5 (45.25–83.75)	69.5 (51.0–88.0)	0.230
CRP ≥ 10 (%)	239 (81%)	119 (77.3%)	120 (85.1%)	0.102
Procalcitonin	0.27 (0.11–0.71)	0.28 (0.10–0.66)	0.27 (0.11–0.79)	0.612
Glutamyltranspeptidase (U/L)	16.0 (11.0–39.75)	17.0 (11.6–40.0)	13.1 (10.1–39.0)	0.100
Alanine transaminase (IU/L)	21.6 (13.2–38.12)	23.7 (14.0–38.9)	19.8 (12.65–36.85)	0.406
Aspartate aminotransferase (IU/L)	29.00 (22.65–40.95)	29.9 (22.8–41.4)	26.7 (22.0–40.6)	0.269
AST/ALT	1.45 (0.97–2.13)	1.45 (0.94–2.08)	1.45 (1.01–2.15)	0.750
Total bile acid (umol/L)	5.70 (3.20–10.93)	5.30 (3.20–10.30)	5.70 (3.20–11.8)	0.361
Total bilirubin (umol/L)	3.85 (2.50–6.00)	3.40 (2.30–5.20)	4.3 (2.9–6.95)	0.007
Albumin (g/L)	39.90 (37.10–42.70)	39.4 (35.8–42.2)	40.2 (37.65–43.00)	0.053
Uric acid (umol)	195.8 (150.5–244.3)	202.0 (154.0–254.0)	189.0 (147.0–236.2)	0.250
Creatinine (umol/L)	26.00 (22.00–31.95)	25.1 (22.0–31.0)	26.0 (22.15–32.0)	0.406
Serum sodium (mmol/L)	138.3 (136.6–139.8)	138.1 (136.28–139.9)	138.3 (136.8–139.6)	0.856
Fibrinogen (g/L)	5.57 (3.95–6.86)	5.13 (3.82–6.84)	5.89 (4.36–6.94)	0.156
Activated partial thromboplastin time (s)	29.6 (27.2–32.15)	29.5 (27.25–31.75)	29.6 (27.0–32.55)	0.615

*CALs* coronary artery lesions, *CI* confidence interval, *IVIG* intravenous immunoglobulin, *RDW-CV* Red blood cell distribution width variation coefficient, *RDW-SD* Red blood cell distribution width standard deviation, *CRP* C-reactive protein, *AST* Aspartate aminotransferase, *ALT* Alanine transaminase

### Independent Risks for Predicting CALs by Multivariate Analysis

To evaluate the independent effects of RDW on CALs in patients with KD, we performed a multivariate logistic analysis. In patients with complete KD, a high RDW-SD was associated with CALs after adjusting for age, sex, hemoglobin, glutamyltranspeptidase, procalcitonin, AST/ALT, albumin, serum sodium, RDW-CV and IVIG resistance [odds ratio 1.115 (95% confidence interval 1.046–1.188); *p* = 0.001] (Table 3). The multivariate logistic regression model also showed that age [odds ratio 1. 066 (95% confidence interval 1.000–1.012); *p* = 0.050], male sex [odds ratio 0.669 (95% confidence interval 0.503–0.888); *p* = 0.006], and hemoglobin(odds ratio 0.974 [95% confidence interval 0.958–0.991]; *p* = 0.003) were closely associated with CALs (Table 3). After adjusting for age, sex, mean erythrocyte volume and total bilirubin, the high RDW-CV levels was an independent risk factor for the development of CALs in patients with incomplete KD [odds ratio 1.428 (95% confidence interval 1.056–1.930); *p* = 0.021] (Table 3).

**Table 3:** Logistic regression analysis of Kawasaki disease with CALs

KD type	Variables	OR	95% CI	*p* value
Complete Kawasaki disease	Age	1.006	1.000–1.012	0.050
	Male sex	0.669	0.503–0.888	0.006
	IVIG resistance	1.661	0.795–3.469	0.177
	Hemoglobin	0.974	0.958–0.991	0.003
	Glutamyltranspeptidase	1.000	0.999–1.001	0.965
	Procalcitonin	1.007	0.981–1.034	0.609
	Albumin	0.971	0.938–1.001	0.099
	Serum sodium	0.960	0.916–1.007	0.093
	AST/ALT	0.975	0.885–1.075	0.610
	RDW-CV	1.078	0.894–1.301	0.432
	RDW-SD	1.115	1.046–1.188	0.001
Incomplete Kawasaki disease	Age	1.003	0.994–1.013	0.464
	Male sex	0.634	0.385–1.046	0.074
	Mean erythrocyte volume	0.977	0.917–1.042	0.485
	Total bilirubin	0.931	0.861	1.007
	RDW-CV	1.428	1.056–1.930	0.021

*CALs* coronary artery lesions, *CI* confidence interval, *IVIG* intravenous immunoglobulin, *OR* odds ratio, *RDW-CV* Red blood cell distribution width variation coefficient, *RDW-SD* Red blood cell distribution width standard deviation

### ROC Analysis

**Fig. 1:**
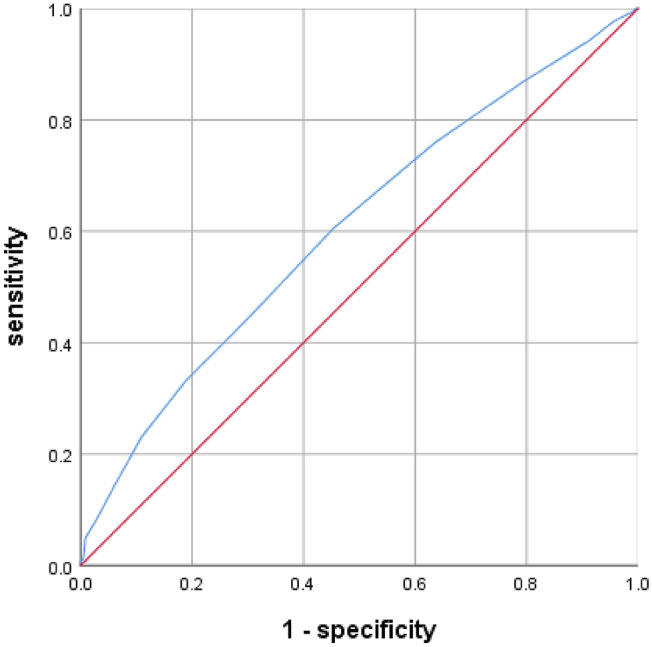
Receiving Operating Characteristic (ROC) of Red blood cell distribution width standard deviation (RDW-SD) for predicting coronary artery lesions in patients with complete Kawasaki disease

**Fig. 2:**
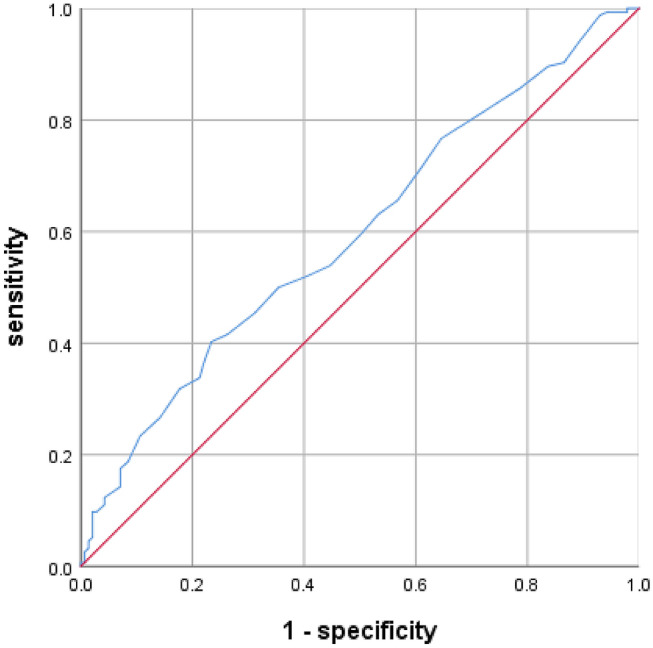
Receiving Operating Characteristic (ROC) of Red blood cell distribution width variation coefficient (RDW-CV) for predicting coronary artery lesions in patients with incomplete Kawasaki disease

According to the ROC curve analysis, the optimal cut-off value of RDW-SD in patients with complete KD for predicting CALs was 38.5 fL, with a sensitivity of 61% and a specificity of 55% (AUC was 0.606, 95% confidence interval 0.572–0.640; *p* = 0.000; Fig. 1). The optimal cut-off value of RDW-CV in patients with incomplete KD for predicting coronary artery lesions was 13.85, with a sensitivity of 40% and a specificity of 77% (AUC was 0.597, 95% confidence interval 0.532–0.661; *p* = 0.004; Fig. 2).

## Discussion

The present study demonstrated that a high RDW-SD is an independent marker of CALs in patients with complete KD. We found this association to be independent of multiple potential confounding factors, including age, sex, IVIG resistant, hemoglobin, RDW-CV, glutamyltranspeptidase, procalcitonin, AST/ALT, albumin, and serum sodium. Additionally, the AUC of RDW-SD for the predictor of CALs in patients with complete KD was 0.606 (95% confidence interval 0.572–0.640; *p* = 0.000), with a sensitivity and a specificity value of 61% and 55%, respectively, when the optimal cut-off value of RDW-SD was 38.5 fL. While in patients with incomplete KD, we found that a high RDW-CV is an independent marker of CALs after adjusting for age, sex, mean erythrocyte volume and total bilirubin. The AUC of RDW-CV for the predictor of CALs in patients with incomplete KD was 0.597 (95% confidence interval 0.532–0.661; *p* = 0.004), with a sensitivity and a specificity value of 40% and 77%, respectively, when the optimal cut-off value of RDW-CV was 13.85. To our knowledge, this is the first study to evaluate the association between RDW-SD and CALs in patients with KD.

RDW is routinely measured in clinical practice as part of the CBC. RDW is often expressed as RDW-CV and RDW-SD; RDW-SD has better sensitivity and is less affected by other factors compared with RDW-CV [19]. However, studies conducted so far only consider the role of RDW-CV as a predictor of CALs in patients with KD. Xu et al. [20] based their study on 422 patients with KD who were followed up for a period of 3 months. They observed that a baseline RDW-CV above 14.55% is an independent predictor of CALs (odds ratio 3.422, 95% confidence interval 2.014–6.045, *p* = 0.000). Similarly, Zhu et al. [21] revealed that an elevated RDW-CV value of > 13.35% is associated with CALs in patients with KD. Unfortunately, in our study, RDW-CV is only an independent risk factor for CALs in patients with incomplete KD. In patients with complete KD, although univariate analyses showed that RDW-CV is a significant predictor of CALs, significance was not retained with multivariate logistic regression. Compared with studies by Xu et al. and Zhu et al., we controlled for additional confounding factors, including age, sex and hemoglobin. In our study, RDW-SD is an independent predictor of CALs in patients with complete KD. RDW-SD may therefore outperform RDW-CV as a predictor of CALs in patients with complete KD.

RDW values are typically elevated under conditions of ineffective RBC production, increased RBC destruction, and blood transfusion. Nevertheless, the pathophysiological path of increased RDW in patients with KD with CALs remains unknown. Although the precise mechanism by which elevated RDW is associated with CALs in patients with KD is beyond the scope of this study, several explanations are possible. One of the possible mechanisms is inflammation, which plays an important role in the process of KD. In the acute phase of KD, the levels of various inflammatory cytokines, such as tumor necrosis factor-α, interleukin (IL)-1, IL-2, and IL-6, are relatively high and contribute to CAL formation [22–25]. Demir et al. [26] found that patients with KD and CALs have more severe inflammation compared with patients without CALs; thus, markers such as neutrophil–lymphocyte ratio, neutrophil count, and C-reactive protein, differed significantly in this population. Inflammatory states are strongly related to ineffective erythropoiesis, and the release of inflammatory cytokines may increase anisocytosis by suppressing erythropoietin activity and inhibiting erythrocyte maturation [27]. Neurohormonal activation may also contribute to an increase in RDW in patients with KD and CALs. N-terminal pro-brain natriuretic peptide (NT-proBNP), which is an important index of the neurohormonal pathway, is a valuable biomarker for predicting CALs in patients with KD [28, 29]. In our study, we did not measure NT-proBNP; however, Fukuta et al. [30] found that even after adjusting for numerous potential confounders, elevated BNP levels were independently associated with a higher RDW in patients with coronary artery disease.

Additionally, Kim et al. [31] demonstrated that indicators of iron deficiency anemia, especially ferritin, were highly associated with coronary artery abnormalities. Kuo et al. [32] showed that inflammation-induced hepcidin is associated with anemia development and outcomes in patients with KD. High levels of hepcidin lead to a low serum iron content and limit availability of iron for erythropoiesis, resulting in an increase in RDW [33]. However, no indices reflecting iron metabolism were available in our study; therefore, we were unable to confirm a relationship between RDW and iron metabolism. We found that patients with KD and CALs had a lower mean erythrocyte volume, which may indirectly reflect iron deficiency. Thus, impaired iron mobilization may be a pathophysiological mechanism of the association between increased RDW and CALs.

Nonetheless, several limitations hindered our study. First, this was a retrospective study; therefore, selection bias may exist. Second, due to lack of height data, CALs were assessed according to absolute coronary artery diameter rather than mean BSA-adjusted Z-score. In addition, due to the limited sample size, we did not conduct a subgroup analysis of CAL severity. Third, despite adjusting for multiple variables, it is possible that other residual confounding variables influenced the results, such as iron, folate, and vitamin B12. Finally, although an association between an increase in RDW and presence of CALs in patients with KD was established in the present study, whether RDW has a causal role in CAL development or whether it acts as a marker of the disease process needs further investigation.

## Conclusion

In conclusion, RDW-SD is an independent predictor of CALs in patients with complete KD, but this relationship is not observed for incomplete KD. On the contrary, the RDW-CV is an independent predictor of CALs in patients with incomplete KD, but not in complete KD. Therefore, we propose that future studies on the correlation between RDW and KD should pay attention to the differences in the types of KD and attach importance to the value of RDW-SD, which does not include MCV in the calculation. Our findings call for further studies to achieve a better understanding of the mechanism by which RDW relates to KD.

## Data Availability

The data and material are available from the corresponding author on reasonable request.

## Abbreviations

CALs: Coronary artery lesions
KD: Kawasaki disease
RDW: Red blood cell distribution width
RDW-SD: Red blood cell distribution width standard deviation
RDW-CV: Red blood cell distribution width coefficient of variation
